# A Robust Adaptive Unscented Kalman Filter for Nonlinear Estimation with Uncertain Noise Covariance

**DOI:** 10.3390/s18030808

**Published:** 2018-03-07

**Authors:** Binqi Zheng, Pengcheng Fu, Baoqing Li, Xiaobing Yuan

**Affiliations:** 1 Science and Technology on Microsystem Laboratory, Shanghai Institute of Microsystem and Information Technology, Chinese Academy of Sciences, Shanghai 201800, China; bqzheng@mail.sim.ac.cn (B.Z.); fupc@mail.sim.ac.cn (P.F.); sinoiot@mail.sim.ac.cn (B.L.); 2 University of Chinese Academy of Sciences, Beijing 100049, China

**Keywords:** Adaptive filter, data fusion, robust state estimation, nonlinear system, uncertain noise covariance

## Abstract

The Unscented Kalman filter (UKF) may suffer from performance degradation and even divergence while mismatch between the noise distribution assumed as a priori by users and the actual ones in a real nonlinear system. To resolve this problem, this paper proposes a robust adaptive UKF (RAUKF) to improve the accuracy and robustness of state estimation with uncertain noise covariance. More specifically, at each timestep, a standard UKF will be implemented first to obtain the state estimations using the new acquired measurement data. Then an online fault-detection mechanism is adopted to judge if it is necessary to update current noise covariance. If necessary, innovation-based method and residual-based method are used to calculate the estimations of current noise covariance of process and measurement, respectively. By utilizing a weighting factor, the filter will combine the last noise covariance matrices with the estimations as the new noise covariance matrices. Finally, the state estimations will be corrected according to the new noise covariance matrices and previous state estimations. Compared with the standard UKF and other adaptive UKF algorithms, RAUKF converges faster to the actual noise covariance and thus achieves a better performance in terms of robustness, accuracy, and computation for nonlinear estimation with uncertain noise covariance, which is demonstrated by the simulation results.

## 1. Introduction

Accurately and timely estimating the dynamic state in a nonlinear system is an important research area and has attracted considerable interest. For decades, many classic nonlinear estimation algorithms such as the extended Kalman filter (EKF) [1], unscented Kalman filter (UKF) [2], particle filter (PF) [3] and a large number of their varieties are put forward to address the estimation of dynamic state in nonlinear system. Among them, UKF is a considerably typical nonlinear filter algorithm, which has many merits such as simplicity in realization, high accuracy, and rapid convergence [4,5]. Consequently, it has been applied to many areas such as target tracking, navigation, energy estimation, structural dynamics, system identification, and vehicle positioning [6,7,8,9,10,11].

However, UKF as one of Kalman filter varieties is also the minimum mean square error (MMSE) estimator. It requires that the priori statistical characteristics of system noise be precisely known. Among these statistical characteristics, the process noise covariance and the measurement noise covariance are the most important as they directly regulate the impact of prediction values and measurements on the system state estimations [12,13]. However, in practical applications, it is difficult to accurately describe covariances, which may suddenly change, leading to biased or even divergent filter solutions. Therefore, it is absolutely necessary to adaptively update the two noise covariances by using the information obtained in the filter process [14].

Recently, many efficient adaptive filter algorithms are proposed to address the above problem, which are usually divided into four categories: Bayesian, maximum likelihood (ML), correlation, and covariance-matching methods. Among the four categories, the covariance-matching method is the most computationally efficient [15]. Over the decade, many covariance-matching adaptive filter algorithms were proposed to address the above noise mismatch problem such as multiple model-based [7,16], innovation-based or residual-based [17,18,19] adaptive estimation algorithms. In [20], the authors propose an online adaptive UKF to update noise covariance matrices by minimizing a cost function built based on the errors between the covariance matrices of innovation and their corresponding estimations. However, considering a great amount of derivative calculations involved in the minimization process, this method is difficult to achieve the real-time performance. Shi et al. [21] adopt a modified Sage-Husa noise statistics estimator to estimate and adaptively adjust the process noise covariance, which is able to compensate the errors resulting from the change of noise statistics.

To obtain an accurate noise covariance, the Sage windowing method is frequently used to estimate system noise statistics based on windowing approximation [15]. For example, in [22], the authors present an adaptive UKF by combining the windowing and random weighting concepts and then extend the windowing method to nonlinear system. Reference [19] performs correction for the noise covariance depending on the type of the fault and also uses a windowing method when updating the process noise covariance. In [23], the authors propose an adaptive unscented particle filter which focuses on the adaptive estimation of the measurement noise covariance by using a windowing method with a fixed window size. Nevertheless, the sliding window method acquires the estimation of the current noise covariance by smoothing the relevant parameters during previous timesteps, which not only brings in heavy computational burden but also cannot adapt to the rapid changes of the system state.

Instead of using the windowing method, another approach to adjust noise covariance is to scale the noise covariance matrix with a time dependent variable. For example, in [24] a novel adaptive UKF algorithm is employed to estimate the attitudes of a pico satellite, in which the process noise covariance is adapted by a scaling factor calculated from the residual sequences. Furthermore, the authors propose a fault-detection method and the process noise covariance is only corrected when the fault occurs. In [25] the same authors put forward another adaptive UKF for the same purpose, where the measurement noise covariance is scaled by a scaling matrix instead of the process noise covariance, at the presence of measurement malfunction. Li et al. [26] presents a robust Masreliez–Martin UKF which can provide reliable state estimates in the presence of both unknown process noise and measurement noise covariance matrices. This method also adopts a fading factor to adjust the pre-designed process noise covariance. Nevertheless, the positive definite is not guaranteed in this scaling method [27]. Because the scaling factor is usually obtained by subtracting the two sum of traces of positive define matrices. Thus, in some actual situations, the scaling factor may be negative, and then the new coverance matrix will not be a positive definiteness matrix. Additionally, the calculation of scale factors is also complex and unsuitable for devices with limited computational power, e.g., low-priced sensors.

In this paper, a robust adaptive UKF (RAUKF) is proposed for nonlinear state estimation with uncertain noise covariance to adaptively adjust the noise covariance according to its current estimation and previous value. Note that the uncertainty here contains two aspects: (i) the unknown of actual noise covariance in the estimation; and (ii) the dynamic change of actual noise covariance during the estimation. More specifically, at each timestep, a standard UKF will be implemented first to obtain current state estimations using the new acquired measurement. Then an online fault-detection mechanism is performed by using a statistical function to judge whether the current noise covariance needs to be updated. If needed, the innovation-based and residual-based methods are used to calculate the estimations of process and measurement noise covariance, respectively. Furthermore, by utilizing a weighting factor, the filter obtains the new priori noise covariance values via combining the last ones with their current theoretical estimations. Finally, the state estimations will be corrected according to these new noise covariance matrices and previous state estimations. Unlike the above existing adaptive UKF algorithms based on the windowing or the scale factor, RAUKF need neither to store and smooth the relevant parameters during previous timesteps nor calculate scale factor at each timestep, which brings about a noteworthy reduction in the computational burden. Therefore, it is particularly well adapted for use in devices with limited computational power. Moreover, the proposed RAUKF algorithm could ensure that the new prior covariance matrices are certainly positive definite matrices, since it updates its covariance matrices with a weighting sum of two positive definite matrices. Simulation results show that RAUKF performs well in accuracy, robustness, and computation with uncertain noise covariance.

The rest of this paper is structured as follows. In Section 2, we introduce the nonlinear state estimation based on standard UKF (SUKF). Section 3 describes the proposed RAUKF algorithm. The simulation results of the proposed algorithm is investigated and evaluated in Section 4. Finally, Section 5 gives some conclusions to this paper.

## 2. Nonlinear State Estimation Based on SUKF

As we all know, the Kalman filter relies on two models, namely: a system or process model that describes the state of transition and it is used for prediction; and a measurement model that describes the relationship between state and measurements. In this paper, the state of system evolves according to the following discrete-time dynamic model:

(1) $$x_{k}=f\left(x_{k-1}\right)+w_{k-1},$$

where *k* is the sampling time index, f(∗) is the state transition function, $x_{k}$ is the state vector at timestep *k*, and $w_{k-1}$ is the process noise vector, assumed the zero-mean white Gaussian with covariance matrix $Q_{k-1}$. The corresponding error covariance matrix of state $x_{k}$ is defined as

(2) $$P_{k}^{xx}=E\left[\left(x_{k}-{\hat{x}}_{k|k}\right){\left(x_{k}-{\hat{x}}_{k|k}\right)}^{T}\right],$$

where ${\hat{x}}_{k|k}$ is the state estimation at timestep *k*, E(∗) is the expectation operation.

Subsequently, the measurements of system state at each timestep can be given indirectly by the following discrete observation model:

(3) $$z_{k}=h\left(x_{k}\right)+v_{k},$$

where h(∗) is the measurement function, $z_{k}$ is the measurement at timestep *k*, and $v_{k}$ is the measurement noise vector, which is also considered to be Gaussian with zero mean and covariance matrix $R_{k}$.

With respect to the two system models, the nonlinear estimation based on SUKF can be briefly expressed as follows [2,6]. Note that the initial state vector ${\hat{x}}_{0}$ and its error covariance matrix ${\hat{P}}_{0}$ are assumed to be known and the initial noise covariance matrices $Q_{0}$ and $R_{1}$ are set to the positive definite matrices by users.

1. Compute weights with the initial parameter $0<\omega_{0}<1$:
  (4) $$\omega_{j}=\frac{\left(1-\omega_{0}\right)}{2n_{x}},j=1,\cdots ,2n_{x}$$
  (5) $$\;c^{j}=\sqrt{\frac{n_{x}}{1-\omega_{0}}}r^{j},j=1,\cdots ,2n_{x},$$
  where $n_{x}$ is the dimension of the state vector, $\{r^{j};j=1,\cdots ,n_{x}$} is the *j*th standard basis vector and $r^{j}=-r^{\left(j-n_{x}\right)}$ when $j=n_{x}+1,\cdots ,2n_{x}$. How to select an appreciate $\omega_{0}$ is also a meaningful topic and readers could refer to reference [2,28] for more details about the selection of $\omega_{0}$.
2. At timestep *k*, establish symmetric sigma points ϕ at timestep k−1 with the last state estimation ${\hat{x}}_{k-1|k-1}$ and last estimation of state error covariance ${\hat{P}}_{k-1|k-1}^{xx}$ which are both assumed to have been obtained from the previous step:
  (6) $$\;\;D_{k-1|k-1}={\left({\hat{P}}_{k-1|k-1}^{xx}\right)}^{1/2}$$
  (7) $$\phi_{k-1|k-1}^{\left(0\right)}={\hat{x}}_{k-1|k-1},\;\;\phi_{k-1|k-1}^{\left(j\right)}={\hat{x}}_{k-1|k-1}+D_{k-1|k-1}c^{j},j=1,\cdots ,2n_{x}.$$
3. Predict the current states ${\bar{x}}_{k|k-1}$ and its error covariance matrix ${\bar{P}}_{k|k-1}^{xx}$:
  (8) $${\bar{x}}_{k|k-1}=\sum_{j=0}^{2n_{x}}\omega_{j}f\left(\phi_{k-1|k-1}^{\left(j\right)}\right)$$
  (9) $$\begin{array}{c} {\bar{P}}_{k|k-1}^{xx}=\sum_{j=0}^{2n_{x}}\omega_{j}[f\left(\phi_{k-1|k-1}^{\left(j\right)}\right)-{\bar{x}}_{k|k-1}]\times {\left[f\left(\phi_{k-1|k-1}^{\left(j\right)}\right)-{\bar{x}}_{k|k-1}\right]}^{T}+Q_{k-1}. \end{array}$$
4. Establish symmetric sigma points ϕ about the state prediction:
  (10) $$\;\;D_{k|k-1}={\left({\hat{P}}_{k-1|k-1}^{xx}\right)}^{1/2}$$
  (11) $$\phi_{k|k-1}^{\left(0\right)}={\bar{x}}_{k|k-1},\;\;\phi_{k|k-1}^{\left(j\right)}={\bar{x}}_{k|k-1}+D_{k|k-1}c^{\left(j\right)},j=1,\cdots ,2n_{x}.$$
5. Define $\mu_{k}=z_{k}-h\left({\bar{x}}_{k|k-1}\right)$ as the innovation vector at timestep *k* and the innovation covariance matrix is $P_{k}^{zz}=E\left[\mu_{k}*{\left(\mu_{k}\right)}^{T}\right]$. Then, the predicted innovation covariance matrix ${\bar{P}}_{k|k-1}^{zz}$ and cross covariance matrix ${\bar{P}}_{k|k-1}^{xz}$ are separately determined by:
  (12) $$\begin{array}{c} {\bar{P}}_{k|k-1}^{zz}=\sum_{j=0}^{2n_{x}}\omega_{j}\left[h\left(\phi_{k|k-1}^{\left(j\right)}\right)-{\bar{z}}_{k|k-1}\right]\times {\left[h\left(\phi_{k|k-1}^{\left(j\right)}\right)-{\bar{z}}_{k|k-1}\right]}^{T}+R_{k} \end{array}$$
  (13) $$\begin{array}{c} {\bar{P}}_{k|k-1}^{xz}=\sum_{j=0}^{2n_{x}}\omega_{j}[f\left(\phi_{k-1|k-1}^{\left(j\right)}\right)-{\bar{x}}_{k|k-1}]\times {\left[h\left(\phi_{k|k-1}^{\left(j\right)}\right)-{\bar{z}}_{k|k-1}\right]}^{T}, \end{array}$$
  where ${\bar{z}}_{k|k-1}=\sum_{j=0}^{2n_{x}}\omega_{j}h\left(\phi_{k|k-1}^{\left(j\right)}\right)$.
6. Calculate Kalman gain $K_{k}$ and then obtain the estimation of current state ${\hat{x}}_{k|k}$ and its error covariance matrix ${\hat{P}}_{k|k}^{xx}$ with current actual measurement $z_{k}$:
  (14) $$K_{k}\overset{\Delta }{=}{\bar{P}}_{k|k-1}^{xz}{\left({\bar{P}}_{k|k-1}^{zz}\right)}^{-1}$$
  (15) $${\hat{x}}_{k|k}={\bar{x}}_{k|k-1}+K_{k}\left(z_{k}-{\bar{z}}_{k|k-1}\right)$$
  (16) $${\hat{P}}_{k|k}^{xx}={\bar{P}}_{k|k-1}^{xx}-K_{k}{\bar{P}}_{k|k-1}^{zz}{\left(K_{k}\right)}^{T}.$$

To run the SUKF, users need to provide $Q_{k-1}$ in Equation (9) and $R_{k}$ in Equation (12). Performance of a Kalman filter depends on how well users can configure the $Q_{k-1}$ and $R_{k}$ for current applications. Conventionally, they are often configured as constant matrices during the running of SUKF using a trial-and-error approach, which relies on the experience and background of users. Therefore, it is a challenge for users to configure initial $Q_{0}$ and $R_{1}$ at the beginning.

## 3. Robust Adaptive Unscented Kalman Filter

The SUKF algorithm works well under suitable Q and R. However, when users configure unsuitable noise covariance matrices as a priori or there is a fault either in system as a change in the noise covariance as a malfunction, SUKF may fail and thus its estimation results become inaccurate [19].

To address this challenge, we propose a robust adaptive unscented Kalman filter (RAUKF) algorithm. The algorithm adaptively adjusts Q and R based on the weighting combination of their current theoretical estimation values and the last data if a fault occurs in system. Note that SUKF works well in normal cases. Therefore, a fault-detection mechanism should be adopted to judge if it is necessary to revise noise covariance matrices. The adopted fault-detection mechanism in this letter is similar to that used in work [19] which uses the following statistical function to detect the fault:

(17) $$\varphi_{k}=\mu_{k}^{T}{\left[{\bar{P}}_{k|k-1}^{zz}\right]}^{-1}\mu_{k},$$

The $\varphi_{k}$ has a $\chi^{2}$ distribution with *s* degree of freedom and *s* is the dimension of the $\mu_{k}$. Suppose we wish to detect a fault with a reliability level of 1−σ, where σ is a chosen parameter. Then there is a threshold, $\chi_{\sigma ,s}^{2}$, determined by the $\chi_{\sigma ,s}^{2}$ distribution of $\varphi_{k}$ such that

(18) $$P(\varphi_{k}>\chi_{\sigma ,s}^{2})=\sigma$$

Thus, using the preselected value of σ, we define a corresponding fault detection threshold $\chi_{\sigma ,s}^{2}$ from Equation (18), such that a system fault can be detected with a reliability level of 1−σ.

### 3.1. Adaptive Adjustment of Q

The innovation sequence $\mu_{k}$ represents the additional information available to the filter as a consequence of the incoming new measurements. Hence, it is considered as the most relevant information for the filter adaptation and can be used to estimate the noise covariance [23]. According to Equation (1), the process noise can be represented as $w_{k-1}=x_{k}-f\left(x_{k-1}\right)$.

Furthermore, from equations in SUKF, it yields

(19) $$\begin{array}{c} {\hat{w}}_{k-1}={\hat{x}}_{k}-f\left({\hat{x}}_{k-1|k-1}\right)\approx {\hat{x}}_{k}-{\bar{x}}_{k|k-1}\\ \;\;\;=K_{k}\left(z_{k}-{\bar{z}}_{k|k-1}\right)\approx K_{k}\mu_{k}. \end{array}$$

Note that there are two approximations via linearization used in the above derivation, ${\bar{x}}_{k|k-1}\approx f\left({\hat{x}}_{k-1|k-1}\right)$ and ${\bar{z}}_{k|k-1}\approx h\left({\bar{x}}_{k|k-1}\right)$, in which the forward evolution of the mean at time k−1 is approximated by the mean computed from the forward evolved sigma points; respectively the observation of the mean is approximated by the mean observation, computed via the observations of the sigma points.

Therefore, the estimation of process noise covariance matrix $Q_{k-1}$ can be estimated as:

(20) $$Q_{k-1}=cov\left({\hat{w}}_{k-1}\right)=K_{k}E\left[\mu_{k}\mu_{k}^{T}\right]K_{k}^{T},$$

where cov(∗) is the covariance operation and E(∗) is the expectation operation.

To implement above equation, $E(\mu_{k}\mu_{k}^{T})$ usually approximated by means of averaging $\mu_{k}\mu_{k}^{T}$ over time using a windowing method. Instead of using moving window methods (like in works [15,19,23,29]), this paper adaptively adjusts Q by utilizing a weighting factor λ to balance the last noise covariance value and current estimation. The weighting factor λ is set with a lower limit $\lambda_{0}\in \left(0,1\right)$ to ensure the update strength and will increase as the rise of the $\varphi_{k}$ if the $\varphi_{k}$ exceeds a preset threshold. Therefore, the system process noise covariance matrix is updated as:

(21) $$Q_{k-1}=\left(1-\lambda \right)Q_{k-1}+\lambda \left(K_{k}\mu_{k}\mu_{k}^{T}K_{k}^{T}\right),$$

(22) $$\lambda =max\{\lambda_{0},\left(\varphi_{k}-a\times \chi_{\sigma ,s}^{2}\right)/\varphi_{k}\},$$

where a(a>0) is a tuning parameter dependent on the actual environment, and a larger *a* implies a higher probability of using the default $\lambda_{0}$. Specially, how the trade-off between $\lambda_{0}$ and *a* will determine how sensitive the covariance update is to the new innovation statistics.

### 3.2. Adaptive Adjustment of R

The measurement noise covariance matrix R at timestep *k* can also be estimated by the innovation-based approach as follows:

(23) $${\hat{R}}_{k}=E\left[\mu_{k}\mu_{k}^{T}\right]-{\bar{S}}_{k|k-1}^{zz},$$

where ${\bar{S}}_{k|k-1}^{zz}=\sum_{j=0}^{2n_{x}}\omega_{j}\left[h\left(\phi_{k|k-1}^{\left(j\right)}\right)-{\bar{z}}_{k|k-1}\right]\times {\left[h\left(\phi_{k|k-1}^{\left(j\right)}\right)-{\bar{z}}_{k|k-1}\right]}^{T}$. Readers could refer to reference [30] for more details about the derivation process. Generally speaking, the $R_{k}$ should be positive definite as it is a covariance matrix. However, its estimation ${\hat{R}}_{k}$ from Equation (23) cannot guarantee to be a positive definite matrix, since it is obtained by subtracting the two positive definite matrix. Then, if there are negative parameters in the main diagonal of noise covariance matrix, the adaptive filter will suddenly diverge [22]. To obtain a positive definite matrix ${\hat{R}}_{k}$, a residual-based approach is used. From Equation (3) the measurement noise at timestep *k* can be derived as $v_{k}=z_{k}-h\left(x_{k}\right)$. Then, the residual vector at timestep *k* is given by

(24) $$\varepsilon_{k}=z_{k}-h\left({\hat{x}}_{k|k}\right).$$

Furthermore, according to work [23], the estimation of measurement noise covariance based on the residual vector $\varepsilon_{k}$ is given by

(25) $$\begin{array}{c} {\hat{R}}_{k}=cov\left({\hat{v}}_{k}\right)=E\left[\varepsilon_{k}{\varepsilon_{k}}^{T}\right]+{\hat{S}}_{k|k}^{zz}, \end{array}$$

where

(26) $${\hat{S}}_{k|k}^{zz}=\sum_{j=0}^{2n_{x}}\omega_{j}\left[h\left(\phi_{k|k}^{\left(j\right)}\right)-{\hat{z}}_{k|k}\right]\times {\left[h\left(\phi_{k|k}^{\left(j\right)}\right)-{\hat{z}}_{k|k}\right]}^{T},$$

(27) $$\phi_{k|k}^{\left(0\right)}={\hat{x}}_{k|k},\;\phi_{k|k}^{\left(j\right)}={\hat{x}}_{k|k}+D_{k|k}c^{\left(j\right)},j=1,\cdots ,2n_{x},\;D_{k|k}={\left({\hat{P}}_{k|k}^{xx}\right)}^{1/2},$$

(28) $${\hat{z}}_{k|k}=\sum_{j=0}^{2n_{x}}\omega_{j}h\left(\phi_{k|k}^{\left(j\right)}\right).$$

Similar to the method used in the previous section, the $R_{k}$ is updated utilizing a weighting factor δ set with a lower limit $\delta_{0}\in \left(0,1\right)$ as:

(29) $$R_{k}=\left(1-\delta \right)R_{k}+\delta \left[\varepsilon_{k}{\varepsilon_{k}}^{T}+{\hat{S}}_{k|k}^{zz}\right]$$

(30) $$\delta =max\{\delta_{0},\left(\varphi_{k}-b\times \chi_{\sigma ,s}^{2}\right)/\varphi_{k}\},$$

where b(b>0) is also a tuning parameter dependent on the actual environment, and the selection of the *b* and $\delta_{0}$ is the same as that of the *a* and $\lambda_{0}$.

### 3.3. Correct Estimations

Once the covariance matrices $Q_{k-1}$ and $R_{k}$ are updated, current state estimations should be corrected with the new $R_{k},Q_{k-1}$ and the estimations ${\hat{x}}_{k|k}$, ${\hat{P}}_{k|k}^{xx}$ to obtain more accurate state estimations. The correction process is described as follows:

1. Compute the new predicted error covariance matrix ${\bar{P}}_{k|k}^{xx}$ and cross covariance matrix ${\bar{P}}_{k|k}^{xz}$, which treats the acquired state ${\hat{x}}_{k|k}$ as the predicted state of timestep *k*:
  (31) $${\bar{P}}_{k|k}^{xx}=\sum_{j=0}^{2n_{x}}\omega_{j}{\left[\phi_{k|k}^{\left(j\right)}-{\hat{x}}_{k|k}\right]}^{T}\times \left[\phi_{k|k}^{\left(j\right)}-{\hat{x}}_{k|k}\right]+Q_{k-1},$$
  (32) $${\bar{P}}_{k|k}^{xz}=\sum_{j=0}^{2n_{x}}\omega_{j}[\phi_{k|k}^{\left(j\right)}-{\hat{x}}_{k|k}]\times {\left[h\left(\phi_{k|k}^{\left(j\right)}\right)-{\hat{z}}_{k|k}\right]}^{T}$$
2. Evaluate the updated innovation covariance matrix ${\bar{P}}_{k|k}^{zz}$ and Kalman gain ${\hat{K}}_{k}$:
  (33) $${\hat{P}}_{k|k}^{zz}={\hat{S}}_{k|k}^{zz}+R_{k},\;{\hat{K}}_{k}\overset{\Delta }{=}{\bar{P}}_{k|k}^{xz}{\left({\hat{P}}_{k|k}^{zz}\right)}^{-1}$$
3. Correct the estimation of current state ${\hat{x}}_{k|k}$ and its error covariance matrix ${\hat{P}}_{k|k}^{xx}$
  (34) $${\hat{x}}_{k|k}={\hat{x}}_{k|k}+{\hat{K}}_{k}\left(z_{k}-{\hat{z}}_{k|k}\right)$$
  (35) $${\hat{P}}_{k|k}^{xx}={\bar{P}}_{k|k}^{xx}-{\hat{K}}_{k}{\bar{P}}_{k|k}^{xz}{\left({\hat{K}}_{k}\right)}^{T}.$$

In this subsection, we use the estimation ${\hat{x}}_{k|k}$ and its sigma point $\phi_{k|k}^{\left(j\right)}$ to replace the previous forecast ${\bar{x}}_{k|k-1}$ and its sigma point $\phi_{k|k-1}^{\left(j\right)}$ as the new input values in the correction phase. Although this method will incorporate the previous mis-estimated $Q_{k-1}$ and $R_{k}$ into the final estimations, the previous estimation ${\hat{x}}_{k|k}$ obtained from Equation (15) is more precise than the prediction value ${\bar{x}}_{k|k-1}$. Thus, in this paper we adopt the estimation ${\hat{x}}_{k|k}$ and its sigma point $\phi_{k|k}^{\left(j\right)}$ as the input values of our correction phase, which is like in many iterated UKF algorithms, e.g., [31,32].

From Equation (21) and Equation (29), we can find that the proposed RAUKF adopts a one-step update instead of using the windowing procedure, which brings about the decrease of computation burden and is friendly to the sensors with limited computation power and storage capacity. Moreover, the new noise covariance matrices obtained via weighting sum of previous values and current estimation are certainly the positive definite matrices. The proof is given as follows. The overall procedure of the proposed RAUKF algorithm is summarized in Algorithm 1.

**Theorem** **1.** *The new obtained prior noise covariance matrices in Equations (21) and Equation (29) are both the positive definite matrices.*

**Proof.**  At the begaining of the proposed algorithm, the initial noise covariance matrices $Q_{0}$ and $R_{1}$ is set to the positive definite matrices by users. At timestep *k*, the prior noise covariance matrices will be updated if the system fault occurs. The new noise covariance matrices are obtained via weighting sum of their previous values and current estimation which are described in Equations (21) and Equation (29). In the equations, the previous noise covariance matrices $Q_{k-1}$ and $R_{k}$ are already both the positive definite matrices. The expression $K_{k}\mu_{k}\mu_{k}^{T}K_{k}^{T}$ in Equation (21) is a positive definite matrix as it is the product of $\left(K_{k}\mu_{k}\right)$ and its transpose ${\left(K_{k}\mu_{k}\right)}^{T}$. Similarly, the expression $\varepsilon_{k}{\varepsilon_{k}}^{T}$ in Equation (29) is also a positive definite matrix. Furthermore, from Equation 26, the new obtained ${\hat{S}}_{k|k}^{zz}$ is a weighting sum of products which are all positive definite matrices. Thus, it is also a positive definite matrix. Additionly, the weight factors λ,δ∈(0,1). Therefore, the new obtained prior noise covariance matrices $Q_{k-1}$ and $R_{k}$ in Equations (21) and (29) are both the positive definite matrices. ☐

**Algorithm 1.** The robust adaptive unscented Kalman filter (RAUKF) algorithm.**Input:**
$f\left(*\right),h\left(*\right),{\hat{x}}_{0},Q_{0},R_{1},{\hat{P}}_{0},\lambda_{0},\delta_{0},\omega_{0},\chi_{\sigma ,s}^{2}$.1: Initialization:2:       $\omega_{j}=\left(1-\omega_{0}\right)/2n_{x}$, $c^{0}=\sqrt{\frac{n_{x}}{1-\omega_{0}}}$, $c^{\left(j\right)}=\sqrt{\frac{n_{x}}{1-\omega_{0}}}r^{\left(j\right)},j=1,\cdots ,2n_{x}$.3: **for**
k=1→K
**do**4:       Implement SUKF to obtain ${\hat{x}}_{k|k}$, ${\bar{P}}_{k|k-1}^{zz}$, $K_{k}$, ${\hat{P}}_{k|k}^{xx}$.5:       Perform the fault-detection mechanism:6:       $\varphi_{k}=\mu_{k}^{T}{\left[{\bar{P}}_{k|k-1}^{zz}+R_{k}\right]}^{-1}\mu_{k}$7:       **if**
$\varphi_{k}>\chi_{\sigma ,s}^{2}$, **then**8:          Update the $Q_{k-1}$ and $R_{k}$:9:             $\mu_{k}=z_{k}-h\left({\bar{x}}_{k|k-1}\right),\varepsilon_{k}=z_{k}-h\left({\hat{x}}_{k|k}\right)$;10:             ${\hat{S}}_{k|k}^{zz}=\sum_{j=0}^{2n_{x}}\omega_{j}\left[h\left(\phi_{k|k}^{\left(j\right)}\right)-{\bar{z}}_{k|k}\right]\times {\left[h\left(\phi_{k|k}^{\left(j\right)}\right)-{\bar{z}}_{k|k}\right]}^{T}$;11:             $\delta =max\{\delta_{0},\left(\varphi_{k}-a\times \chi_{\sigma ,s}^{2}\right)/\varphi_{k}\}$; $\lambda =max\{\lambda_{0},\left(\varphi_{k}-b\times \chi_{\sigma ,s}^{2}\right)/\varphi_{k}\}$;12:             $Q_{k-1}\leftarrow \left(1-\lambda \right)Q_{k-1}+\lambda \left(K_{k}\mu_{k}\mu_{k}^{T}K_{k}^{T}\right)$; $R_{k}\leftarrow \left(1-\delta \right)R_{k}+\delta \left[\varepsilon_{k}{\left(\varepsilon_{k}\right)}^{T}+{\hat{S}}_{k|k}^{zz}\right]$.13:       Correct state estimations:14:             ${\hat{K}}_{k}\overset{\Delta }{=}{\bar{P}}_{k|k}^{xz}{\left({\hat{P}}_{k|k}^{zz}\right)}^{-1}$;15:             ${\hat{x}}_{k|k}={\hat{x}}_{k|k}+{\hat{K}}_{k}\left(z_{k}-{\hat{z}}_{k|k}\right)$; ${\hat{P}}_{k|k}^{xx}={\bar{P}}_{k|k}^{xx}-{\hat{K}}_{k}{\bar{P}}_{k|k}^{xz}{\left(K_{k}\right)}^{T}$;16:      **end if**17:      $Q_{k}\leftarrow Q_{k-1}$, $R_{k+1}\leftarrow R_{k}$.18:      Save the ${\hat{x}}_{k|k}$ and ${\hat{P}}_{k|k}^{xx}$.19: **end for**

## 4. Simulation Results

To highlight the merits of RAUKF, we conduct the simulation experiment on a simple vehicle-tracking problem in two different cases. In the first experiment, the actual noise covariance matrices are assumed unknown and impossible to estimate off-line. In the second experiment, the actual noise covariance of the system will dynamically change during the estimation. The experiment results are compared with that of SUKF algorithm with fixed noise covariance, AUKF algorithm with a moving window method (called AUKF-window, the window size is set to 10 here) [23], and AUKF algorithm with scaling factor method (called AUKF-scaling-factor) [24]. Note that the AUKF-scaling-factor method in [24] only updates its process noise covariance matrix when the fault occurs. Thus, this method will only be compared with the proposed RAUKF in the second case. All simulations are executed on an Intel Core i5-2520M CPU 2.50 GHz × 4 personal computer with 10 GB of random-access memory.

$x_{k}={\left[x_{k},{\dot{x}}_{k},y_{k},{\dot{y}}_{k}\right]}^{T}$ denotes the vehicle state at discrete timestep *k*, where $p_{k}=\left(x_{k},y_{k}\right)$ is the current vehicle position and $\left({\dot{x}}_{k},{\dot{y}}_{k}\right)$ is its velocity. Δ is the constant sampling time interval between two successive measurements. Meanwhile, a stationary radar is assumed to be used to track the vehicle at position (0,0). Its measurement vector is described by $z_{k}=\left[\rho_{k},\theta_{k},\nu_{k}\right]$, where $\rho_{k}$ and $\theta_{k}$ are respectively the distance and angle between the radar and vehicle, $\nu_{k}$ is the vehicle speed. Therefore, the system models are given by

(36) $$\left\{\begin{array}{c} x_{k}=f\left(x_{k-1}\right)+w_{k-1}=Ax_{k-1}+w_{k-1}\\ z_{k}=h\left(x_{k}\right)+v_{k}=\left[\begin{array}{c} \sqrt{x_{k}^{2}+y_{k}^{2}}\\ \arctan(y_{k}/x_{k})\\ \sqrt{{\dot{x}}_{k}^{2}+{\dot{y}}_{k}^{2}} \end{array}\right]+v_{k}, \end{array}\right.$$

where A is the state transition matrix.

In this paper, the root mean-squared error in position at each timestep, $RMSE_{p}$, and its average, $ARMSE_{p}$, are adopted as the indications of tracking accuracy, since it yields a combined measurement of the bias and variance of a filter estimate [33,34]. The $ARMSE_{p}$ is given by

(37) $$ARMSE_{p}=\sqrt{\frac{1}{N_{m}K}\sum_{i=1}^{N_{m}}\sum_{k=1}^{K}\left[{\left({\hat{x}}_{k}^{\left(i\right)}-x_{k}\right)}^{2}+{\left({\hat{y}}_{k}^{\left(i\right)}-y_{k}\right)}^{2}\right]},$$

and the $RMSE_{p}$ at timestep *k* yields

(38) $$RMSE_{p}\left(k\right)=\sqrt{\frac{1}{N_{m}}\sum_{i=1}^{N_{m}}\left[{\left({\hat{x}}_{k}^{\left(i\right)}-x_{k}\right)}^{2}+{\left({\hat{y}}_{k}^{\left(i\right)}-y_{k}\right)}^{2}\right]},$$

where $\left({\hat{x}}_{k}^{\left(i\right)},{\hat{y}}_{k}^{\left(i\right)}\right)$ is the estimated vehicle position in timestep *k* at *i*-th Monte Carlo run. $N_{m}$ is the number of Monte Carlo runs and K=100 is the number of time sample in one run. $\chi_{\sigma ,s}^{2}$ is taken as 2.37, and this value comes from chi-square distribution when the degree of freedom is 3 and the reliability level is 50%. Other parameters in the simulations are set as follows:
$Q^{\circ }=9*\left[\begin{array}{cccc} \frac{\Delta^{3}}{3} & \frac{\Delta^{2}}{2} & 0 & 0\\ \frac{\Delta^{2}}{2} & \Delta & 0 & 0\\ 0 & 0 & \frac{\Delta^{3}}{3} & \frac{\Delta^{2}}{2}\\ 0 & 0 & \frac{\Delta^{2}}{2} & \Delta \end{array}\right]$,$A=\left[\begin{array}{cccc} 1 & \Delta & 0 & 0\\ 0 & 1 & 0 & 0\\ 0 & 0 & 1 & \Delta \\ 0 & 0 & 0 & 1 \end{array}\right]$,$R^{\circ }=diag\left(\left[1,0.0001,9\right]\right)$,${\hat{P}}_{0}=diag\left(\left[2,3,2,3\right]\right)$,${\hat{x}}_{0}=\left[0,10,0,10\right]$,Δ=0.1,$N_{m}\;=\;1000$,$\lambda_{0}=\delta_{0}=0.2$,
where $Q^{\circ }$ and $R^{\circ }$ are the actual process and measurement noise covariance matrices of our simulation system, respectively.

### 4.1. Selection of Tuning Parameters a and b

Before implementing the simulation experiments, we need to select suitable tuning parameter *a* and *b*. According to Equations (21), (22) and (29), (30), the adaptive adjustments of $Q_{k-1}$ and $R_{k}$ have the same theories and structures. Thus, in this paper, the parameter *a* and *b* are given the same values.

To select suitable values for the two tuning parameters, they are varied from 1 to 8 in two different situations. In first situation, the initial prior noise covariance matrices are set to the actual noise covariance matrices, namely, $Q_{0}=Q^{\circ },R_{1}=R^{\circ }$. While, the initial prior noise covariance matrices in another one is that $Q_{0}=100\times Q^{\circ },R_{1}=0.01\times R^{\circ }$.

**Figure 1:**
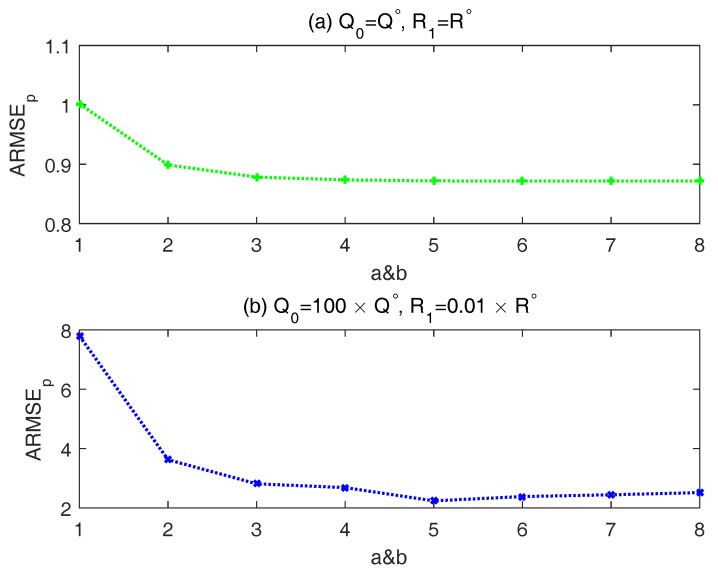
$ARMSE_{p}$ results of different weighting parameters in two different situations.

As shown in Figure 1a, the improvement of estimation accuracy diminishes and seems to be trivial after a>4. In this situation, the prior noise covariance is close to the actual one. Thus, the weighting factor λ should be as small as possible and then a large *a* implies that the λ will be set to its lower limit $\lambda_{0}$ with a very high probability. From Figure 1b, the $ARMSE_{p}$ decreases first and then rising a little. In this situation, a moderate tuning parameter is necessary, as too large or too small tuning parameter may have negative influences on the covariance-matching. The principle of selecting δ is the same with that of λ. Therefore, in the following simulation experiments, *a* and *b* are chosen as 5.

### 4.2. Simulation Results in Unknown Noise Environment

In the first simulation case, we evaluate the performance of RAUKF with arbitrary initial noise covariance matrices. Specifically, $Q_{0}$ and $R_{1}$ are varied apart from small to large by scaling $Q^{\circ }$ and $R^{\circ }$ to test the accuracy and robustness of RAUKF. Under the same setup of simulations, the $ARMSE_{p}$ results of different algorithms are respectively summarized from Table 1, Table 2 and Table 3.

**Figure 2:**
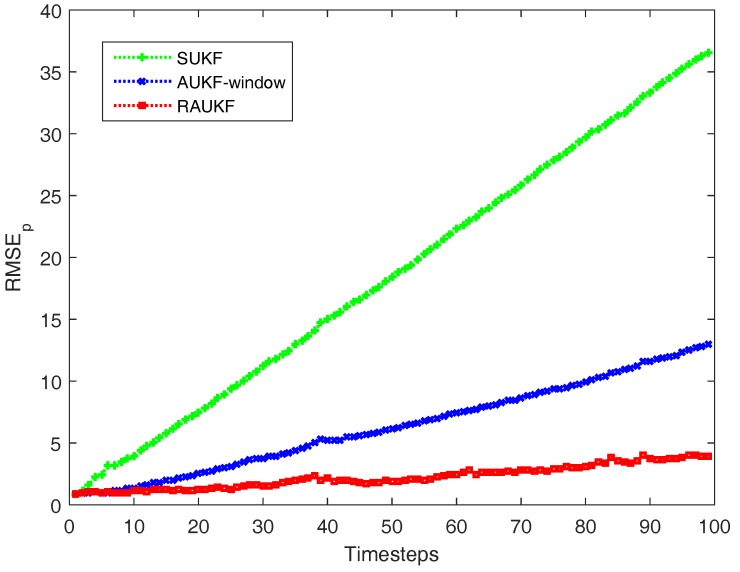
Comparison of $RMSE_{p}\left(k\right)$ results of different algorithms when $Q_{0}=100\times Q^{\circ }$ and $R_{1}\;=\;0.01\;\times \;R^{\circ }$.

Comparing the three tables, one can observe that in general the proposed RAUKF achieves the best performance in robustness and accuracy among the three algorithms. In detail, from Table 1, the $ARMSE_{p}$ results of SUKF are relatively greater when the $Q_{0}$ or $R_{1}$ is set to a large or small value than those of a normal range, especially in the situation that the initial noise covariance matrices are considerably lower than the actual ones. In many cases, the $ARMSE_{p}$ results are more robust in AUKF-window rather than in SUKF by contrasting Table 1 and Table 2. However, owing to the window smoothing operations, the $ARMSE_{p}$ results of AUKF-window are almost similar in most of different initial noise covariance matrices. Furthermore, they are slightly above that of SUKF except at the bottom left of the table. It says that the windowing-based AUKF improves the robustness of estimation but reduces its accuracy. Meanwhile, in some cases (e.g., m = 0.01, n = 100) the $ARMSE_{p}$ results are also large. As shown in Table 3, the performance improvement of RAUKF is more obvious when the $ARMSE_{p}$ results of AUKF-window and SUKF are both large. For example, Figure 2 shows the $RMSE_{p}$ results of the three algorithms at each timestep when $Q_{0}=100\times Q^{\circ }$ and $R_{1}=0.01\times R^{\circ }$. Additionally, in the normal situations, the $ARMSE_{p}$ results of RAUKF are also almost the same as those of SUKF. In summarize, RAUKF achieves a good performance even if users know very little about the actual noise covariance and uses an unsuitable approximation.

As for the computational performance, all algorithms are run in the same computer and their running time results are all averaged over 1000 runs. The time consumption of RAUKF is 28.40 ms for each run, which is slightly higher than that of SUKF (23.30 ms). This is because RAUKF needs to update the noise covariance matrices and correct the state estimations. However, AUKF-window’s computation time is 52.04 ms, which is much greater than that of SUKF and RAUKF. This is due to it involves a vast number of calculations in the updates of noise covariance utilizing a windowing method. Furthermore, the length of the window in AUKF-window determines the amount of the measurements that need to be accumulated and averaged to calculate the estimated covariance matrices [23]. Thus, the longer the length of the window is, the higher the computation is.

### 4.3. Simulation Results in Dynamic Noise Environment

In the second case, we assume that the actual noise intensity of the vehicle occurs a sudden dramatic change during the tracking to highlight the robustness of RAUKF. Therefore, the actual process noise covariance in this simulation is set as

(39) $$\left\{\begin{array}{c} Q_{k}^{\left(v\right)}=Q^{\circ },k=1,\cdots ,20\\ Q_{k}^{\left(v\right)}=100\times Q^{\circ },k=21,\cdots ,K. \end{array}\right.$$

It should be noted that the parameter δ is set as 0, since the $R_{k}^{\left(v\right)}$ of the vehicle do not change during the simulation. In this case, the proposed RAUKF algorithm will be compared with AUKF-window, AUKF-scaling-factor, and SUKF for a comprehensive evaluation. Meanwhile, the $R_{k}$ of AUKF-window keeps fixed for fairness to compare with RAUKF. $R_{1}=R^{\circ }$ and other parameters are the same with previous setup.

**Figure 3:**
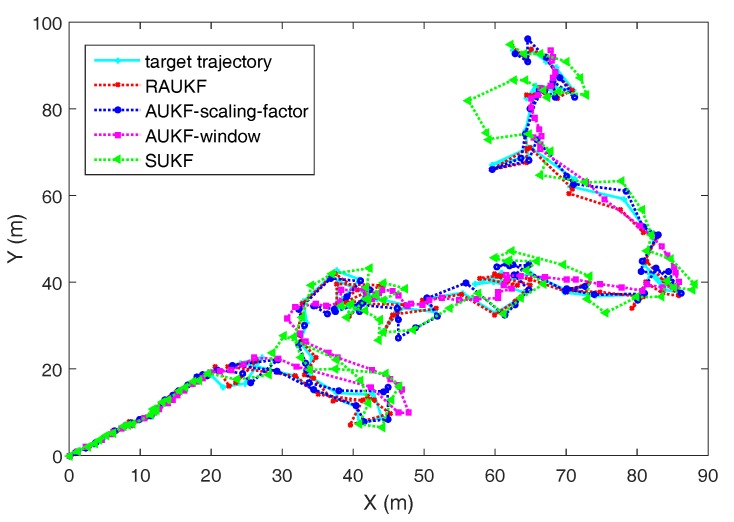
Comparison of state estimation results of different algorithms before and after an abrupt change of the $Q_{k}^{\left(v\right)}$.

**Figure 4:**
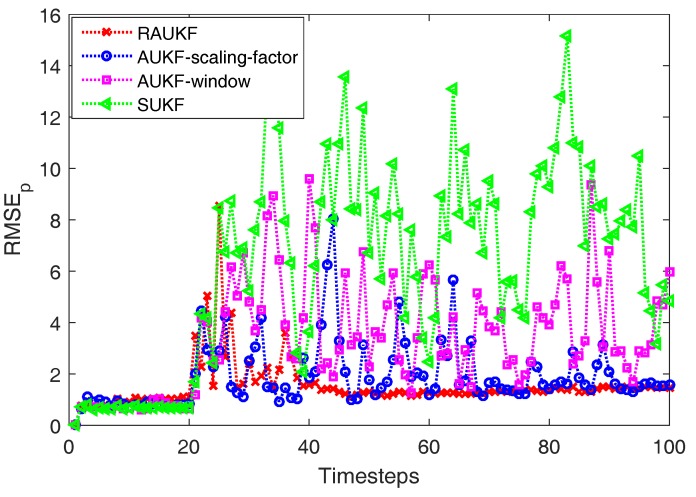
Comparison of $RMSE_{p}\left(k\right)$ results of different algorithms before and after an abrupt change of the $Q_{k}^{\left(v\right)}$.

Figure 3 shows one of comparison results of different algorithms in state estimation at each timestep and Figure 4 presents the $RMSE_{p}$ results of the four algorithms at each timestep, from which we can see that RAUKF successfully track the vehicle after an abrupt change of the $Q_{k}^{\left(v\right)}$. Moreover, SUKF cannot track the vehicle well now due to the fixed preudonoise intensity, which couldn’t provide enough drive power to the related estimation. Thus, its $RMSE_{p}$ results increase rapidly after timestep 20. As for the comparison of AUKF-window, AUKF-scaling-factor, and RAUKF, their $Q_{k}$ will increase after timestep 20 because of the adaptive adjusting mechanisms. Thus, the $RMSE_{p}$ results of them are all steady decline after timestep 20. However, the proposed RAUKF performs better than that of AUKF-window method and AUKF-scaling-factor method.

More concretely, as shown in Table 4, the averaged $RMSE_{p}$ results of the four different algorithms before timestep 20 are almost the same, which means that all of them could deal with the normal situation well. Then, the actual process noise of system changes at the timestep 20, the averaged $RMSE_{p}$ results of the four different algorithms begin to be different. There is no doubt that SUKF algorithm performs the worst among the four algorithms after timestep 20 as the mismatch between its prior noise covariance and the actual one. The averaged $RMSE_{p}$ of the proposed RAUKF after timestep 20 is the lowest compared with that of AUKF-window method and AUKF-scaling-factor method, which shows the superiority of RAUKF in dealing with the nonlinear estimation in dynamic noise environment. In detail, the convergence speed of $RMSE_{p}$ of RAUKF is the fastest among the three different adaptive algorithms (about 10 sample steps).

## 5. Conclusions

In this paper, a robust adaptive unscented Kalman filter (RAUKF) is proposed for the state estimation of nonlinear system under a sequential framework with uncertain noise covariance. A fault-detection mechanism is used to judge if it is necessary to update the noise covariance matrices. If necessary, RAUKF will adjust Q and R based on the weighting combination of current theoretical estimation and previous values. The state estimations will then be corrected. Unlike many existing adaptive UKF algorithms based on the windowing or the scale factor, RAUKF need neither to store and smooth the relevant parameters during previous timesteps nor calculate scale factor at each timestep, which brings about a noteworthy reduction in the computational burden. Therefore, it is particularly well adapted for use in devices with limited computational power. Moreover, the proposed RAUKF algorithm could ensure that the new prior covariance matrices are certainly positive definite matrices, since it updates its covariance matrices with a weighting sum of two positive definite matrices. Simulations results from two aspects demonstrate that the proposed RAUKF performs well in accuracy and robustness on nonlinear estimation with uncertain noise covariance.

This work adaptively updates the process and measurement noise covariance simultaneously when the system fault is detected. Thus, in the further research work, we will focus on investigating how to exactly detect the type of the fault (either a process noise uncertainty or a measurement malfunction). Then the process noise covariance or measurement noise covariance will be revised respectively according to the fault type.

## Figures and Tables

**Table 1:** $ARMSE_{p}$ results of SUKF.

${\mathrm{ARMSE}}_{p}$		$R_{1}=m\times R^{\circ }$				
		m = 0.01	m = 0.1	m = 1	m = 10	m = 100
$Q_{0}=n\times Q^{\circ }$	n = 0.01	7.1569	5.0975	1.0752	1.2882	1.4639
	n = 0.1	7.1716	5.0995	0.8666	1.0588	1.2598
	n = 1	7.1879	5.1180	0.8563	0.8496	1.0433
	n = 10	7.1974	5.0640	0.9520	0.8692	0.9305
	n = 100	21.3282	18.1290	1.0739	1.3955	2.7678

**Table 2:** $ARMSE_{p}$ results of AUKF-window (windowsize = 10).

${\mathrm{ARMSE}}_{p}$		$R_{1}=m\times R^{\circ }$				
		m = 0.01	m = 0.1	m = 1	m = 10	m = 100
$Q_{0}=n\times Q^{\circ }$	n = 0.01	1.6672	1.6577	1.6136	1.5858	1.5636
	n = 0.1	1.6558	1.6561	1.6128	1.5851	1.5534
	n = 1	1.6629	1.6470	1.6054	1.5791	1.5613
	n = 10	5.0705	1.6141	1.5831	1.5578	1.5492
	n = 100	7.2723	6.6321	1.6130	1.5886	1.5847

**Table 3:** $ARMSE_{p}$ results of RAUKF.

${\mathrm{ARMSE}}_{p}$		$R_{1}=m\times R^{\circ }$				
		m = 0.01	m = 0.1	m = 1	m = 10	m = 100
$Q_{0}=n\times Q^{\circ }$	n = 0.01	1.5843	1.4737	1.1664	1.2476	1.4639
	n = 0.1	1.4917	1.3922	1.1271	1.0368	1.2598
	n = 1	1.3424	1.2940	1.0827	0.8472	1.0433
	n = 10	1.8842	1.6269	1.1120	0.8752	0.9305
	n = 100	2.4231	3.7601	1.5362	1.3892	2.5616

**Table 4:** Averaged $RMSE_{p}$ before and after timestep 20.

Averaged ${\mathrm{RMSE}}_{p}$	SUKF	AUKF-Window	AUKF-Scaling-Factor	RAUKF
Before timestep 20	0.6886	0.7397	0.7970	0.8163
After timestep 20	7.6859	4.0566	2.1757	1.6093
